# Mitochondrial Stress in Metabolic Inflammation: Modest Benefits and Full Losses

**DOI:** 10.1155/2022/8803404

**Published:** 2022-11-22

**Authors:** Qing Yuan, Z. L. Zeng, Shiqi Yang, Anqi Li, Xuyu Zu, Jianghua Liu

**Affiliations:** ^1^Department of Metabolism and Endocrinology, The First Affiliated Hospital, Hengyang Medical School, University of South China, Hengyang, China; ^2^Diabetes Clinical Medical Research Center of Hunan Province, Department of Metabolism and Endocrinology, The First Affiliated Hospital, Hengyang Medical School, University of South China, Hengyang, China

## Abstract

Energy intake and metabolic balance are the pillars of health preservation. Overnutrition causes nonspecific, persistently low inflammatory state known as metabolic inflammation. This condition contributes to the pathophysiology of various metabolic disorders, such as atherosclerosis, obesity, diabetes mellitus, and metabolic syndrome. The mitochondria maintain the balance of energy metabolism. Excessive energy stress can lead to mitochondrial dysfunction, which promotes metabolic inflammation. The inflammatory environment further impairs mitochondrial function. Accordingly, excellent organism design keeps the body metabolically healthy in the context of mitochondrial dysfunction, and moderate mitochondrial stress can have a beneficial effect. This review summarises the research progress on the multifaceted characterisation of mitochondrial dysfunction and its role in metabolic inflammation.

## 1. Introduction

According to the World Health Organization, in 2016, more than 650 million adults over the age of 18 were obese, more than 340 million children and adolescents aged 5–19 were overweight or obese, and the number of people with obesity is increasing sharply and showing a trend of low age. This trend is closely related to sedentary, lack of exercise, the pressure of work, and unhealthy diet including high sugar, fat, and salt intake (who.int).

Chronic overnutrition can trigger a low-level, chronic, noncontrollable inflammatory condition known as metabolic inflammation. This condition contributes to a series of metabolic disorders such as obesity, insulin resistance, and atherosclerosis [1]. Metabolic inflammation does not have typical inflammation pathological features but exhibits tissue and multiple organ dysfunctions [1]. Moreover, metabolic inflammatory diseases are frequently accompanied by significant mitochondrial dysfunction, suggesting that the mitochondrion is closely related to the pathophysiology of metabolic inflammation [2].

As the centre of energy metabolism, the mitochondrion supplies energy to the cell through the production of ATP by oxidative phosphorylation (OXPHOS). In addition, mitochondria play a central role in various biological events, such as the citric acid cycle, fatty acid *β*-oxidation and biosynthesis of some metabolites, signal transduction of calcium ions, regulation of apoptosis, and involvement in developmental and aging processes [2]. Mitochondrial dysfunction is a critical feature of metabolic inflammation, but it has not been fully defined, and its interrelationship with metabolic inflammation is not clear. In this review, we discussed the numerous pathways of mitochondrial dysfunction and the vicious loop between mitochondrial dysfunction and metabolic inflammation. We concluded that considering the physiological design of the body itself, mitochondrial dysfunction is not always an upstream event of metabolic inflammation.

## 2. Mitochondria as a Metabolic Centre

Knowledge about the structure and function of mitochondria helps us understand why the mitochondrion is the centre of metabolism. The endosymbiosis theory of mitochondria is widely accepted. The endosymbiosis theory holds that mitochondria are organelles that evolve from primitive bacteria that participate in the endosymbiosis process with protoeukaryotes [3]. From the outside to the inside, the structure of mitochondria includes the outer mitochondrial membrane (OMM), the intermembrane space (IMS), the inner mitochondrial membrane (IMM), and the mitochondrial matrix. The OMM is a barrier to mitochondria, and it is involved in information exchange between organelles, apoptosis, and inflammatory signal transduction and regulation of mitochondrial dynamics [4, 5]. The IMS is a subcompartment with a diameter of approximately 60 nm, in which proteins are extensively involved in the transport and signalling of metabolites, lipids, and metal ions. The IMS also maintains the activity of the respiratory chain by participating in the assembly of the respiratory chain complex [6, 7]. The IMM has a high protein ratio, indicating that the IMM undertakes more important biochemical reactions, including the OXPHOS. The mitochondrial matrix contains many proteins involved in the Krebs cycle, fatty acid *β*-oxidation, and urea cycle. In addition, mitochondrial DNA (mtDNA) is evenly distributed in the mitochondrial matrix, and this structure is tightly related to mitochondrial biogenesis [8, 9].

**Figure 1:**
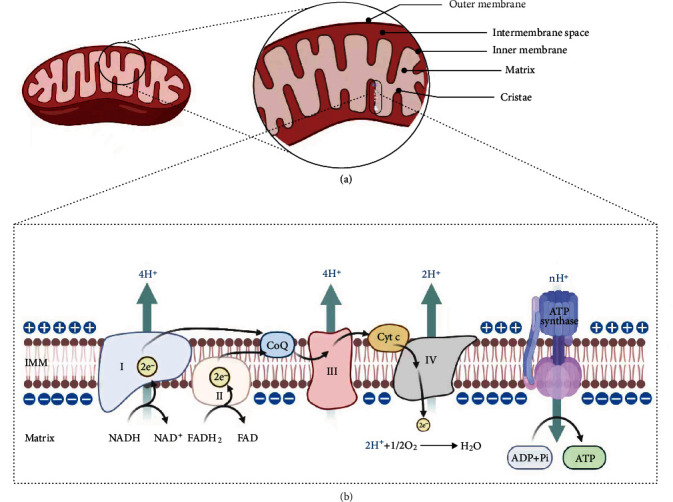
Structure of mitochondria and electron transport chains (ETC). (A) Structure of mitochondria, including OMM, IMS, IMM, and mitochondrial matrix from outside to inside. (B) ETC is a continuous reaction system composed of a series of hydrogen carrier and electron carrier, which accepts the H^+^ removed from metabolites and reduces O_2_ to H_2_O, accompanied by the synthesis of ATP. Created with http://Biorender.com.

As an energy-producing organelle, the mitochondrion is characterised by a highly folded membrane structure, thus providing a large surface area to generate energy. The mitochondrial cristae formed by the highly folded IMM assume the function of OXPHOS. The electron transport chain (ETC) is widely distributed in the mitochondrial crest. This structure consists of four transmembrane protein complexes (Figure 1). Complex I, which is also known as NADH-ubiquinone oxidoreductase or NADH dehydrogenase, is responsible for the transfer of electrons from matrix NADH to ubiquinone. Complex I comprises 44 subunits in mammals and is the largest complex in ETC. Electron microscopy results exhibit that the complex I is “L” shaped, in which the cross arm consists of hydrophobic subunits anchored in the inner membrane and the other arm protruding into the matrix. The matrix arm contains two structural domains, namely, the N module, in which the flavin mononucleotide (FMN) molecule is responsible for accepting electrons from NADH, and the Q module, which has the ubiquinone binding site [10, 11]. Complex I receives electrons from the matrix NADH to FMN and then passes through the Fe/s cluster to ubiquinone. Through the Fe/s prosthetic group of the hydrophobic subunit, the ubiquinone transfers electrons to the ubiquinone of IMM and finally reduces the ubiquinone to ubiquinol (QH2) [10, 11]. Complex II (succinate dehydrogenase) transfers electrons from succinate to ubiquinone. Complex II consists of four subunits, of which two hydrophobic subunits anchor the complex to the IMM and two subunits located on the matrix side contain succinic acid binding sites, Fe/S clusters, and FAD auxiliaries. Complex II receives electrons from succinic acid dehydrogenation and transfers them to ubiquinone to reduce ubiquinol (QH2) without proton transfer [12–14]. Complex III (CoQ-cytochrome C reductase) receives electrons from OH2 to cytochrome C. Complex III is the structure of homodimer, and each unit consists of 11 subunits, in which cytochrome b (bL and bH), cytochrome C1, and Fe/S clusters have redox activity [15]. The electron transport of complex III is achieved through the Q cycle, in which complex III receives electrons from QH2 and transfers them to cytochrome C [16, 17]. Complex IV (cytochrome c oxidase) consists of 13 different subunits, of which four redox centres (hemea, hemea3, CuA, and CuB) constitute two groups of electron transport units (hemea-CuA and hemea3-CuB) [18, 19]. The reduced cytochrome c transfers electrons via CuA to hemea and then to the hemea3-CuB binuclear centre and finally reduces O_2_ to H_2_O [20, 21]. The transmembrane proton gradient associated with the above electron transfer process leads to the conformational change of FoF₁-ATP enzyme, resulting in the phosphorylation of ADP to ATP [22].

The aerobic environment ensures the normal operation of ETC, because O_2_ is the only terminal electron acceptor (TEA), and its reduction is necessary for QH2 oxidation and continuous electron input into ETC. Under physiological conditions, mammalian cells can maintain the normal electron flow of ETC under hypoxia, indicating the presence of potential electron flow pathways that can maintain mitochondrial function under oxygen limitation. Mammalian mitochondria use fumarate as TEA after adapting to O_2_ limitation. The accumulation of OH2 under anoxic conditions can reverse drive succinate dehydrogenase (SDH), resulting in electron deposition on fumarate. Fumarate reduction maintains electron input ETC through complex I and dihydroorotate dehydrogenase (DHODH), but only some tissues have this ability [23]. A typical pathological manifestation of metabolic inflammation is the hypoxia of cells and tissues. The adaptation of ETC in the hypoxia environment provides information for studying diseases with low oxygen levels in tissues (such as obesity and diabetes).

The relationship between mitochondria and metabolism has been widely and profoundly applied. 2,4-Dinitrophenol (DNP), a popular weight-loss drug in the 1930s, blocks mitochondrial ATP synthesis and prompts cells to metabolise carbohydrates and fat, but its production was later discontinued because of severe adverse reactions. However, DNP combined with dichloroacetic acid (DCA, a PDH activator) can alleviate the negative responses of DNP and improve the symptoms of diabetes [24]. In addition, more safe and efficient mitochondrial uncoupling drugs are used in the treatment of metabolic inflammation [25–28]. Considering the abnormal metabolism of tumour cells, targeted mitochondrial antitumour therapy has become a new antitumour direction. Accordingly, Bonekamp et al. [29] designed a highly selective allosteric inhibitor IMT1B, which can affect the normal metabolic function of mitochondria by targeting the inhibition of mitochondrial RNA polymerase activity, thus reducing tumour volume. Similarly, Nuevo-Tapioles et al. [30] found that nebivolol can reduce the energy supply of tumour cells by inhibiting the activity of complex I and ATP enzymes in ETC, thus limiting the growth of tumour cells.

As a metabolic centre, the abundance of mitochondria determines the metabolic level of the organ. Approximately 95% of the heart's energy comes from mitochondria, accounting for one-third of the volume of cardiocyte [31]. The liver has a high number of mitochondria. The liver has the characteristics of metabolic zonation, and the metabolic function and gene expression of hepatocytes in different zonations have significant heterogeneity. By contrast, the morphology and function of the mitochondria, as the critical metabolism sensor, vary according to liver zonation [32]. Mitochondria can regulate systemic metabolism through delivery between different tissues and organs. Brestoff et al. [33] found that macrophages in white adipose tissue (WAT) can obtain mitochondria from neighbouring adipocytes to regulate metabolic homeostasis and maintain energy balance, but the impairment of this transfer mechanism in patients with obesity may reveal the relationship between metabolic imbalance and mitochondria.

## 3. Mitochondrial Dysfunction and Its Multifaceted Character

**Figure 2:**
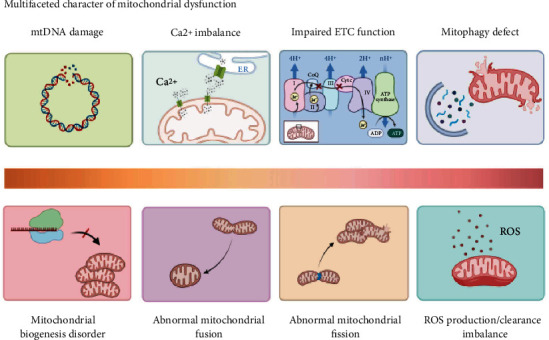
Multifaceted character of mitochondrial dysfunction. This schematic shows the numerous pathways of mitochondrial dysfunction, including mtDNA damage, impaired ETC function, Ca^2+^ imbalance, mitophagy deficiency, mitochondrial biogenesis disorder, abnormal mitochondrial dynamics, and ROS production/clearance imbalance. Created with http://biorender.com.

Mitochondrial dysfunction is the inability of mitochondria to produce ATP levels that maintain the energy needs of cells; that is, the mitochondria cannot fully perform their “energy factory” function. However, mitochondrial dysfunction has multifaceted characteristics, and the mechanism involved is very complex, making it challenging to define mitochondrial dysfunction accurately. Fisher-Wellman et al. [34] developed and verified a multichannel respiratory flux detection platform under multisubstrate and multienergy conditions, which can conduct an in-depth and comprehensive phenotypic analysis of mitochondrial function by simulating the fluctuation of energy supply and demand *in vivo* [34, 35]. This method has been applied to mitochondria from the hearts of mice on a high-fat diet, and the results show that mitochondrial function was impaired regardless of substrate levels [34, 35]. However, the limitation of this detection platform is that it ignores the interaction between mitochondria and other organelles, and it was unable to analyse various pathological events of mitochondrial dysfunction. Mitochondrial DNA damage, ETC function impairment, calcium (Ca^2+^) imbalance, mitophagy deficiency, mitochondrial biogenesis disorder, abnormal mitochondrial dynamics, and imbalance of production/clearance of reactive oxygen species (ROS) constitute mitochondrial dysfunction (Figure 2). The multifaceted characteristics and pathological effects of mitochondrial dysfunction are introduced below.

### 3.1. Mitochondrial DNA (mtDNA) Damage

Mitochondrial DNA is a double-stranded circular molecule composed of 16,569 bases, which are mainly distributed in the mitochondrial matrix near the ETC. Similar to bacterial chromosomes, mtDNA is encapsulated by a series of proteins to form a nucleoid, in which mitochondrial transcription factor A (TFAM) is the main structure of the nucleoid. mtDNA encodes 37 genes, including 13 peptides, 2 ribosomal RNA (12s and 16s), and 22 tRNAs, of which 13 peptides are the core subunits of ETC complex [36]. mtDNA is usually inherited maternally, but some studies have found that mtDNA is inherited biparentally [37].

mtDNA is more vulnerable to damage than nuclear DNA depending on the following unique physiological background of mtDNA: (1) mtDNA is a naked double-stranded molecule that lacks histone protection; (2) mtDNA is in an active redox environment; (3) mtDNA has no introns and is in a dynamic process of replication and transcription; (4) mtDNA lacks accurate damage repairability; and (5) mtDNA is more attractive for the aggregation of lipophilic compounds such as polycyclic aromatic hydrocarbons [38].

Oxidative stress reduces the fidelity of mtDNA replication and inhibits TFAM-mediated mtDNA maintenance, thus impairing mitochondrial energy metabolism and increasing the release of proinflammatory factors [39, 40]. However, the delivery of mitochondrial components (e.g., TFAM and mtDNA) through extracellular vesicles derived from mesenchymal stem cells can reduce target cells' mtDNA damage, thus restoring the mitochondrial function of damaged cells and reducing inflammation [41]. A strong correlation was observed between mitochondrial DNA damage and aging-related phenotype. Mice with mitochondrial polymerase-*γ* (Polg) deficiency and decreased integrity of mitochondrial DNA showed accelerated vascular aging, which could delay vascular aging after restoring mitochondrial DNA copies [42]. In addition, skin wrinkles and hair loss can be improved by repairing mitochondrial DNA [43].

### 3.2. Impaired ETC Function

The biogenesis of electron transport chain involves many steps, including gene expression encoded by nuclear and mtDNA and the coordinated assembly of different subunits and cofactors [16, 44]. The impairment of the stability and activity of ETC will lead to mitochondrial dysfunction. By contrast, the dysfunction of complex and the loss of factors required for complex synthesis and assembly are the main pathological factors for impaired ETC stability and activity [16, 44].

ETC dysfunction causes the toxic accumulation of NADH, an increase in the NADH/NAD+ ratio, and inhibition of various NAD+-dependent metabolic reactions, including glycolysis [45]. As an essential intermediate in the glycolysis pathway, glycerol-3-phosphate (Gro3P) can maintain the dynamic redox balance of NADH/NAD^+^. Enhancing the biosynthesis of Gro3P can antagonise the dysfunction of complex I and alleviate the metabolic crisis, which may provide a new therapeutic target for mitochondrial dysfunction-related diseases caused by the deletion of complex I [45]. Interestingly, a slight decrease in ETC activity during organism development can benefit biological lifespan through epigenetic modification [46]. Decreased ETC activity will reduce the synthesis of metabolite acetyl-CoA (acetyl-CoA). The chromatin remodelling and histone deacetylase complex (NuRD) regulate histone acetylation levels and reshape chromatin structure by responding to acetyl-CoA level. This persistent epigenetic feature will change the progress of aging [47].

### 3.3. Calcium (Ca^2+^) Imbalance

Mitochondria absorb and store calcium ions through calcium ion targets that are abundant on the membrane surface to regulate the dynamic equilibrium of calcium ions [48]. Mitochondrial calcium homeostasis highly affects the function of excitable cells, such as neurons and cardiomyocytes. Low mitochondrial Ca^2+^ uptake will lead to loss of excitable cell function, while calcium overload causes cell injury and apoptosis [48].

Mitochondrial calcium unidirectional transporter (Uniplex) mediates mitochondrial calcium uptake, and this structure is mainly composed of four protein subunits, including MCU (forming ion channels), MICU1, and MICU2 (with gated properties), and accessory subunit EMRE (mediating Ca^2+^ transport) [49]. Cryo-EM observed that at low Ca^2+^, MICU1-MICU2 was fastened to the IMS side surface of MCU-EMRE tetramer similar to a lid, thus preventing the intake of Ca^2+^ [49]. In high-Ca^2+^ conditions, MICU1-MICU2 shifts to the edge of the MCU-EMRE tetramer, allowing Ca^2+^ to pass freely through the MCU channel [49]. EMRE maintains MICU1-MICU2 near MCU and ensures the movement of MICU1-MICU2 when the concentration of Ca^2+^ changes [49].

The normal transmission of calcium signals can ensure the body's nutritional perception, and the changes of mitochondrial calcium flux may affect metabolic inflammation. MCU expression in adipose tissue may increase during obesity and insulin resistance [50]. The increased expression of MCU enhances mitochondrial Ca^2+^ uptake, and continuous calcium overload reduces NAD+/NADH ratio and antioxidant activity, leading to ROS overproduction [51]. The inhibition of mitochondrial calcium overload can combat brown adipose tissue whitening induced by a high-fat diet and prevent abnormal energy storage and metabolic dysfunction [52]. However, impaired MCU-mediated calcium uptake also disrupts metabolism, and the loss of MCU in hepatocytes reduces OXPHOS efficiency and leads to abnormal lipid accumulation [53]. These studies show a dynamic buffer range of mitochondrial calcium concentration, and the mitochondrial dysfunction caused by the loss of this buffering capacity is a pathological event that is involved in the occurrence and development of metabolic inflammation.

### 3.4. Mitochondrial Autophagy Defects

Mitochondrial autophagy (mitophagy) is mainly used for mitochondrial quality control, because it selectively eliminates damaged or inefficient mitochondria to maintain normal mitochondrial metabolic activity. A short-term HFD diet compensatively activates mitophagy in response to metabolic overload; after long-term HFD feeding, the early compensatory mechanism is disrupted by chronic obesity, and mito-DAMPs (“mito-DAMPs”: oxidized mtDNA, dsDNA, dsRNA, cardiolipin, and ROS) can lead to metabolic abnormalities and excessive inflammation [54–56]. However, pharmacological interventions targeting mitophagy can improve metabolic inflammation [57–61].

The PINK1/Parkin is a crucial ubiquitinated mitophagy pathway that is regulated by IMM and OMM synergistically. PINK1 resides on IMM; upon crossing the IMM in normal mitochondria, PINK1 is identified and cleaved by MPP and PARL before being destroyed by proteasome [62]. However, when the mitochondria are damaged, membrane potential depolarisation causes the accumulation of PINK1 on OMM. PINK1 recruits E3 ubiquitin ligase Parkin to initiate mitophagy [62]. The accumulation of damaged mitochondria can exacerbate inflammation and impair the normal function of brown adipocytes. Mice with mitophagy-deficient Parkin^−/−^ and PINK1^−/−^ exhibit an obese phenotype and elevated levels of inflammatory cytokines in the serum [63, 64].

Mitophagy is initiated by recruiting autophagosomes or boosting autophagosome production, which is mediated by the autophagy receptor BNIP3L/Nix on the OMM [65]. Under obese condition, inadequate oxygen is supplied to hypertrophic adipocytes, and high respiration levels in inflamed tissues can trigger HIF1a-coordinated anoxic responses. Hypoxia is the upstream signal that triggers BNIP3L/Nix-mediated mitophagy. BNIP3L-dependent mitophagy triggers the switch for metabolic reprogramming, which is necessary for proinflammatory/M1 macrophage polarisation [66].

### 3.5. Mitochondrial Biogenesis Disorders

Another aspect of mitochondrial quality control is mitochondrial biogenesis, which is similar to the two sides of a coin. Mitochondrial biogenesis is associated with mitophagy, and aberrant mitophagy impedes mitochondrial biogenesis [67]. Mitochondrial biogenesis originates from a preexisting mitochondrial pool, and it requires coordination between mitochondrial and nuclear genomes, including mtDNA transcription and translation and the synthesis, transport, and assembly of nuclear DNA-encoded mitochondrial protein [68]. In addition, MCU-mediated calcium uptake can promote TFAM dephosphorylation to enhance mitochondrial biogenesis [69].

Peroxisome proliferator-activated receptor-*γ* coactivator-1*α* (PGC-1*α*) is the primary mitochondrial biogenetic mediator. PGC-1*α* is also involved in regulating lipid metabolism and the maintenance of metabolic homeostasis [70]. The mitochondrial biogenetic disorder is related to metabolic inflammation. Abnormalities of mitochondrial biogenesis can be observed in obesity, insulin resistance, type 2 diabetes, nonalcoholic fatty liver, and vascular calcification [71–76]. After promoting mitochondrial biogenesis, it can improve the abnormality of lipid metabolism and alleviate disease progression [58, 77–80]. Moderate exercise during pregnancy can also protect the offspring from hepatic steatosis induced by HFD, which depends on mitochondrial biogenesis [81]. The inhibition of mitochondrial biogenesis can restrict the malignant development of tumours following metabolic reprogramming because of the critical function of mitochondrial biogenesis in metabolic control [82].

### 3.6. Abnormal Mitochondrial Dynamics

Mitochondria form a highly plastic dynamic equilibrium network through interconnection, and their fusion and fission are the key events of dynamic balance. Changes in nutrient supply and metabolic requirements affect the balance in the direction of fission or fusion [83–85]. The transcription level of the mitochondrial fusion protein decreases in the pathological background of obesity and insulin resistance, which can be reversed by weight loss [86]. Similarly, decreased expression of the mitochondrial fusion protein (Mfn1 and Mfn2) and increased expression of fission protein (Drp1) have been observed in the WAT of mice with HFD [87]. And metabolic inflammation can be reversed by regulating mitochondrial dynamics [57, 88–91]. For a better understanding of how mitochondrial dynamics are involved in developing metabolic inflammation, the molecular mechanisms of mitochondrial fusion and fission need to be determined.

As double-membrane organelles, Mfn1 and Mfn2 mediate OMM fusion and OPA1 mediates IMM fusion. Mfn-mediated OMM fusion consists of three consecutive steps, namely, tethering, docking, and merger [92]. Mfn2 sustains dimerization via the GTPase domain following GTP hydrolysis, thus enhancing its membrane tethering efficacy. However, mitochondrial fusion requires a constant GTP hydrolysis cycle, and the continual dimerization of Mfn2 reduces the GTP conversion rate; thus, the increased membrane tethering efficiency of Mfn2 may not always result a higher fusion activity, which must be studied further [93]. In addition, the heterodimer formed by Mfn2 and Mfn1 through the GTPase domain has higher fusion activity than Mfn homodimer; therefore, the ratio of Mfn2 to Mfn1 will also affect the fusion process [93, 94]. After OMM completes the fusion, IMM quickly and efficiently follow up to complete the fusion. Optic atrophy 1 (OPA1) is hydrolysed into membrane-anchored long OPA1 (L-OPA1) and soluble short OPA1 (S-OPA1) by protease. L-OPA1 and S-OPA1 work together to ensure the normal fusion of IMM. OPA1 forms three interfaces, namely, “back-to-back,” “head-to-head,” and “head-to-tail” [92]. The instantaneous “head-tail” assembly of OPA1 causes the bending of the membrane and the formation of unstable membrane protuberances on the two opposite IMM. When the membrane protuberances on both sides are docked, IMM fusion can be completed [92]. The “back-to-back” and “head-to-head” interfaces are involved in the shaping of the mitochondrial cristae [92].

Drp1 is an essential protein that induces mitochondrial fission. Under baseline or stimulating settings, dynamic protein (DNM1, DNM2, and DNM3) knockout does not result in mitochondrial fission abnormalities and excessive fusion, which can be easily recognized by knocking out Drp1 [95]. Drp1 serves several purposes, including interaction with Drp1 receptors (e.g., MFF, Mid49, Mid51, and FIS1), posttranslational modification of Drp1, association with actin cytoskeleton or mitochondrial lipid cardiolipins, and interaction with numerous organelles (e.g., endoplasmic reticulum) [96, 97]. Drp1 mediates two distinct forms of fission, namely, midzone fissions and peripheral fissions, each with a unique role and mechanism [98]. No significant difference was observed in the physiological state of mitochondria before and after the midzone fissions, but the level of ROS increased, Ca^2+^ overload occurred, and the membrane potential and pH value in the mitochondria decreased before peripheral fissions [98]. Therefore, peripheral fission may be a precursor to mitophagy initiation by damaged mitochondria. By contrast, midzone fissions lead to mitochondrial proliferation, and the paradox that mitochondrial fission leads to mitochondrial proliferation and degradation seems plausible [98].

Dysregulated mitochondrial dynamics can lead to oxidative stress, and the accumulation of damaged mitochondria can impair the integrity of the mitochondrial network, which can alter the normal mitochondrial responsiveness to metabolic signals. In a research among several ethnic groups, the risk of obesity and diabetes is associated with the expression of mitochondrial dynamics-related proteins [99]. *In vitro* experiments proved that cells induced by high glucose and fat (HG/HF) showed excessive mitochondrial fission and decreased mitochondrial fusion protein expression [100]. Further, in the pathological background of obesity and diabetes, in both humans and mice, a fragmented mitochondrial network and low mitochondrial fusion protein expression level can be observed [101]. Therefore, abnormal mitochondrial dynamics may be a critical pathological event in metabolic inflammation.

### 3.7. ROS Production/Clearance Imbalance

ROS is a by-product produced by the reaction of electrons escaping from OXPHS with oxygen [102]. The production/clearance of ROS maintains the balance of intracellular redox, and the clearance of ROS mainly depends on antioxidant systems, including manganese superoxide dismutase (MnSOD), glutathione and thioredoxin/peroxiredoxin systems located in the mitochondrial matrix, and copper/zinc superoxide dismutase located in the IMS [102]. The physiological levels of ROS are a key redox signal, which mediates physiological processes through the reversible oxidation of cysteine residues, such as signal transduction of cell pathways, redox control of transcription factors (TFs), the regulation of epigenetic modification patterns, energy metabolism and circadian rhythm dynamics, and maintenance of protein homeostasis [102].

When ROS production/clearance imbalance occurs, high levels of ROS induce oxidative stress to damage biological macromolecules (e.g., proteins, DNA, and lipids) and promote the progression of metabolic inflammation. The hypertrophy and proliferation of adipocytes during obesity respond to overnutrition, but the accumulation of adipocytes tends to produce excessive ROS [103]. High levels of ROS exacerbate inflammation by activating the NLRP3 inflammasome, and inflammation worsens obesity and insulin resistance, leading to a vicious circle [104]. Moreover, ROS can induce obesity through adipogenesis and lipogenesis and inhibit weight gain after antioxidant treatment [105]. In addition, obesity can be reversed through dietary intervention and exercise training, depending on improving oxidative stress [60, 79, 80, 106–111].

## 4. Mutual Regulation between Mitochondrial Dysfunction and Metabolic Inflammation

**Figure 3:**
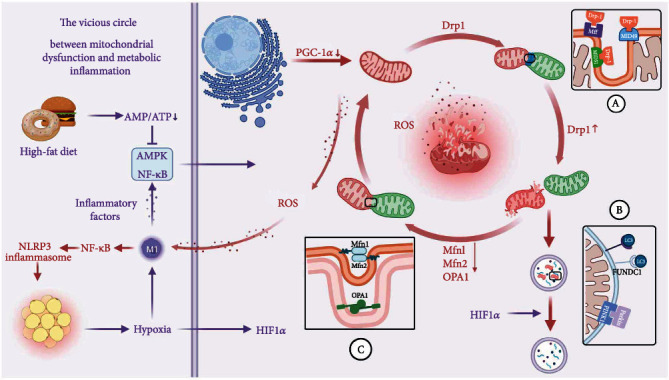
The vicious circle between mitochondrial dysfunction and metabolic inflammation. Overnutrition disrupts energy balance, thus inhibiting AMPK activity, driving the release of inflammatory factors, and thus enhancing NF*κ*B signalling. Metabolic stress and inflammatory signalling disrupt mitochondrial quality control, including excessive mitochondrial fission (A) and fusion defects (C), reduced mitochondrial biosynthesis, and excessive mitophagy (B), which lead to the overproduction of ROS. ROS leads to oxidative stress oxidative stress, including NF*κ*B signalling activation, NLRP3 inflammatory formation, and macrophage M1 polarisation. This condition aggravates the development of metabolic inflammation. Created with http://biorender.com.

Metabolic inflammation is a low-grade, persistent, and progressive inflammation. Overnutrition causes metabolic overload, and the body responds with a series of acute adaptive reactions to preserve equilibrium. Adipocytes increases their lipid storage capacity through hyperplasia to prevent the formation of ectopic fat deposits [112]. By elevating the expression of stress indicators, hypertrophic adipocytes promote the recruitment of proinflammatory macrophages [112]. This specific metabolic stimulation of adipose tissue macrophages (increased OXPHOS and glycolysis) increases inflammatory responses [113]. Moreover, the inflammation of adipocytes can stimulate the growth and remodelling of healthy adipose tissue to store surplus nutrients and induce insulin resistance [114]. Induced insulin resistance is a physiological response to metabolic stress, and it promotes nutritional mobilisation and reduces energy accumulation in cells [115, 116]. The physiological role of insulin resistance during pregnancy supports that insulin resistance reduces maternal glucose intake and promotes maternal lipolysis to meet the nutritional needs of foetal growth [116]. However, this acute adaptive response occurs at the expense of metabolic flexibility, and persistent inflammatory activation will deteriorate the metabolic health of the entire body. A prolonged chronic inflammatory phase will ensue when the metabolic pressure surpasses the threshold for adaptive response. A metabolic energy crisis must be marked by diminished mitochondrial activity. Mitochondrial dysfunction can be noticed in the evolution of metabolic inflammation, which is frequently a vicious cycle (Figure 3).

Overnutrition promotes the dynamic balance of mitochondrial network to mitochondrial fission, which is initially a compensatory mechanism for metabolic stress by increasing the number of mitochondria to meet cellular metabolic demands. However, chronic metabolic stress exacerbates the fragmentation of the mitochondrial network, accompanied by excessive ROS production [117]. This finding is reflected in mice with obesity. The increased Drp1 protein expression and the decreased OPA1 expression were observed in the adipose tissue of Ob/Ob mice [91]. During overnutrition, fragmented mitochondrial networks transform function from OXPHOS to ROS production, which drives macrophage inflammatory polarisation and accumulation of inflammatory signals that exacerbate obesity and insulin resistance [118]. OPA1 promotes autonomous browning of adipocytes, and brown adipose tissue reduces obesity through energy conversion. By contrast, reduced OPA1 expression in overnutrition impairs adipocyte plasticity, thus exacerbating the obesity phenotype [119].

Moreover, excessive substrate supply during obesity will impair mitochondrial ADP sensitivity, increase proinflammatory NF*κ*B signalling, and thus impair mitochondrial respiratory function. By contrast, the decrease in mitochondrial respiratory capacity will drive ROS release [120, 121]. The findings are supported by a research on the correlation between adipocyte size and mitochondrial respiratory capacity, in which the increase in adipocyte diameter was adversely connected with adipocyte mitochondrial respiratory capacity [122]. Impaired mitochondrial biogenesis was also found in patients with obesity in a study of subcutaneous adipose tissue from healthy monozygotic twin pairs [123]. This finding was obtained, possibly because the intracellular milieu of oxidative stress under overnutrition can damage mtDNA and impede mitochondrial synthesis. Consequently, under overnutrition, mitochondrial activity diminishes, the build-up of damaged mitochondria and oxidative stress may produce mitochondrial dysfunction, and mitochondrial dysfunction exacerbates the development of metabolic inflammation.

## 5. Mitochondrial Dysfunction Is Not Always Upstream of Metabolic Inflammation

Numerous research has investigated the alterations in mitochondrial function in the pathogenic context of metabolic inflammation. However, the causal association is hard to demonstrate. Surprisingly, several investigations have discovered that the body may exhibit advantageous metabolism despite the presence of severe mitochondrial dysfunction. TFAM is required to begin mtDNA transcription and maintain mtDNA stability; however, animals with adipose tissue-specific TFAM deletion exhibit favourable metabolic benefits and prevent diet-induced obesity and insulin resistance [124]. Mice with mtDNA mutant showed signs of metabolic imbalance, and high-fat diet reverses the metabolic imbalance and improves the mitochondrial function in these mice [125]. Similarly, mice with PGC1 *α* and PGC1 *β* deficient are still lean mice that are metabolically healthy [126]. Although OPA1 deficiency impairs mitochondrial respiratory capacity, positive metabolic benefits were observed in mice with OPA1 knockout [127]. In addition, mice with ETC proteome imbalance in brown adipose tissue can show a healthy metabolism [128].

In the therapy of metabolic inflammation, the restoration of metabolic balance does not appear to be dependent on mitochondria. Caloric restriction (CR) is the first line of defence against metabolic inflammation, PGC-1 is the primary response factor of CR, and PGC-1 can increase mitochondrial density and oxidation capacity; therefore, the metabolic benefits of CR can be attributed to the stimulation of mitochondrial biogenesis. Finley et al. [129] discovered that mice with skeletal muscle-specific deletion of PGC-1 during CR exhibit adequate metabolic balance. Similar improvements in glucose homeostasis and insulin sensitivity were seen in mice with adipose-specific PGC-1/PGC-1 double deletion during CR [130].

In conclusion, mitochondrial dysfunction and metabolic inflammation seem distinct; that is, mitochondrial dysfunction does not fully converge with the molecular signals that drive metabolic inflammation. This condition may have resulted from the body's complex potential methods for coping with energy crises in the setting of mitochondrial failures, such as the compensation mechanism of metabolic organs or the reprogramming of bioenergetics. These possible therapeutic processes increase FGF21 secretion, mitochondrial oxidation, and thermogenic modulation of WAT and BAT and increase energy expenditure [124–127]. For example, considering the physiological design of the body under energy crises, in a low-energy-intake situation, the body can ensure its blood glucose level through gluconeogenesis (the conversion of metabolic fuel). By looking for the potential mechanism of regulating metabolic homeostasis in mitochondrial dysfunction, it may provide a new direction for treating metabolic inflammation.

## 6. Therapeutic Strategies for Metabolic Inflammation

**Figure 4:**
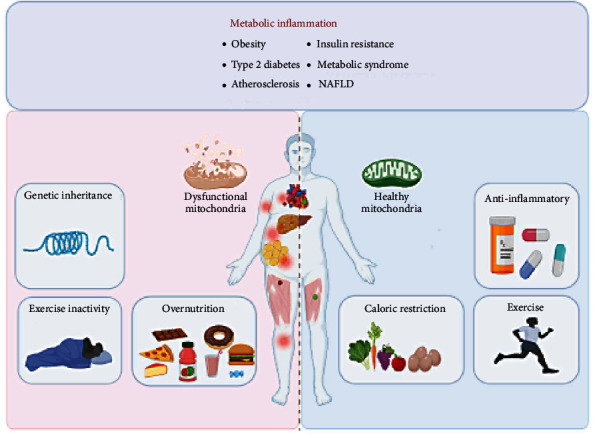
Prevention and treatment of metabolic inflammation. Imbalance in nutritional intake and consumption induces metabolic inflammation (e.g., obesity, IR, TD2M, AS, and NAFLD), while caloric restriction, exercise, and anti-inflammatory therapy can improve metabolic inflammation symptoms and keep mitochondria healthy. Created with http://biorender.com.

According to the aetiology of metabolic inflammation, three preventative and therapeutic strategies are accepted, namely, minimisation of nutrient intake, increasing energy consumption, and lowering inflammation. Therefore, three prevalent methods are caloric restriction, exercise, and anti-inflammatory medication (Figure 4). The role of mitochondria in this process is discussed below.

### 6.1. Caloric Restriction

The first line of defence against metabolic inflammation is caloric restriction. CR can considerably improve systemic metabolic health, including blood lipid, blood pressure and leptin levels, adiponectin concentrations, body fat accumulation, insulin sensitivity, glucose homeostasis, and oxidative stress [131–133]. Although the metabolic benefits of CR do not necessarily depend on the biogenesis of mitochondria, changes in other mitochondrial functions are still involved in the improvement of metabolic homeostasis. AMP-activated protein kinase (AMPK) plays an essential role as an energy sensor in regulating mitochondrial metabolism and dynamics. CR drives mitophagy by activating AMPK, thus reducing the accumulation of damaged mitochondria, and AMPK can maintain the mitochondrial network in a highly interconnected healthy state [134, 135]. CR can prevent the decline of mitochondrial respiratory function, reduce oxidative stress, and protect the integrity of mitochondrial function [136]. These mitochondrial changes in response to CR are beneficial to the improvement of metabolic flexibility and the reduction of inflammation.

Compliance with the CR remains a major obstacle. Long-term persistence is challenging for most individuals. Therefore, it is a potentially viable strategy for locating CR's intended target. Recent analysis of adipose biopsies from participants in the Comprehensive Assessment of Long-term Effects of Reducing Intake of Energy revealed that CR can activate anti-inflammatory pathways and improve adipose tissue metabolism. Results indicate that the phospholipase PLA2G7 is the central transcription program. After knocking down PLA2G7, metabolic dysfunction and inflammatory response were similarly improved in mice, suggesting that altering PLA2G7 may imitate the advantages of CR [137]. In addition, pharmacological mimics of CR (e.g., polyphenols, polyamines, and glycolysis inhibitors) are effective pathways, which can mimic the changes in mitochondrial function of CR (e.g., enhancing mitophagy and reducing oxidative stress) to produce metabolic improvements and anti-inflammatory effects [138, 139]. However, the dose mode and frequency of CR mimetics administration still need further study.

### 6.2. Physical Exercise

Another strategy to deal with overnutrition is changing metabolic efficiency and increasing energy consumption, and exercise is a feasible and economical treatment intervention. Physical activity can remarkably increase the rate of energy consumption, reduce body weight, minimise ectopic lipid accumulation, and promote white fat browning [140, 141]. Moreover, physical exercise can enhance insulin sensitivity, reverse abnormal glucose tolerance, inhibit macrophage infiltration and the release of inflammatory factors, and reduce inflammation, which are essential for improving metabolic abnormalities [140, 142, 143].

Similar to CR, exercise can improve the bioenergy efficiency of mitochondria, which is not difficult to understand. These actions both produce a negative energy balance and have high ATP requirements. The effects of exercise on the structure and function of mitochondria have been determined. Firstly, exercise training improves the respiratory capacity of mitochondria and increases the number, volume, and density of mitochondria in response to high ATP demand [144, 145]. In addition, exercise can reduce the dysfunction of mitochondrial dynamics caused by overnutrition. Exercise can also increase the ratio of fusion/fission protein to form a more fused mitochondrial tubular network, which is characterised by an increase in the expression level of mitochondrial fusion protein (e.g., Mfn1, Mfn2, and OPA1) and a decrease in the expression level of fission protein (e.g., Drp1 and Fis1) [144, 145]. Moreover, exercise can improve oxidative stress and combat ROS production/clearance disorders caused by overnutrition [145]. Theoretically, improved mitochondrial respiration by exercise could lead to increased ROS release, but oxidative stress does not change significantly during exercise because of the effective quenching of ROS by exercise-activated antioxidant systems [146, 147]. However, ROS emission is abnormally reduced after excessive high-intensity exercise, which can be understood as the mitochondrial compensatory shutdown of partial metabolism to reduce overall ROS production to fight oxidative stress and maintain redox homeostasis [146, 148].

The link between exercise and metabolism advantages is not linear. The moderate activity has the most positive influence on metabolic health, whereas excessive exercise has the opposite effect. Excessive high-intensity exercise causes significant mitochondrial respiratory diseases, blood sugar abnormalities, and decreased insulin production, and glucose tolerance does not fully recover during the recovery period [146]. Therefore, choosing a suitable amount of training may be more beneficial to improving metabolic health.

### 6.3. Anti-inflammatory Therapy

Anti-inflammation is the fundamental component in the therapy of metabolic inflammation, and reducing inflammation remarkably improves both obesity and insulin resistance. Dietary restriction and physical activity can be used to reduce inflammation, but they depend on the subjective initiative of the individual, resulting in a less successful therapy than anticipated; thus, pharmacological treatment might be needed. Oxidative stress caused by excessive quantities of ROS mainly causes inflammation, and mitochondria are the primary generator of ROS. Targeting mitochondria with antioxidant medicines appears to be a viable method.

MitoQ is a popular antioxidant that targets mitochondria and has a solid scientific foundation. MitoQ consists of coenzyme Q and lipophilic triphenylphosphonium (TPP) cations rapidly collected in mitochondria under the influence of membrane potential and reduced to the ubiquinone form [149, 150]. MitoQ can decrease serum total cholesterol, triglyceride, and blood glucose levels, minimise fat deposition, and enhance glucose metabolism in rats with metabolic imbalance [150]. MitoQ can also improve insulin signal transduction in rats with obesity and reduce weight gain [151]. In addition, MitoQ inhibits inflammatory signal transduction and decreases the amount of inflammatory cytokines (e.g., TNF-*α* and IL-6) [152].

The key mechanisms for its effect are the reduction of mitochondrial oxidative stress and the improvement of mitochondrial dynamics by MitoQ. MitoQ enters the mitochondria and is adsorbed in the IMM, a design that allows MitoQ to target ROS more precisely. High levels of ROS were observed in mice with a metabolic imbalance model, MitoQ could remarkably reduce ROS levels, and MitoQ treatment showed the same effect in patients with diabetes [150, 153]. In addition, MitoQ supplementation can upregulate the expression of mitochondrial fusion protein (e.g., OPA1, Mfn1, and Mfn2) and downregulate the expression of mitotic protein (e.g., Drp1 and Fis1); maintaining the integrity of mitochondrial network can also prevent the excessive production of ROS [154, 155].

The build-up of TPP in IMM will disrupt normal mitochondrial activity, posing a threat to drug safety [149]. A 28-week animal study found that long-term treatment of MitoQ in healthy mice had no discernible adverse effects on mitochondrial function, gene expression of the endogenous antioxidant system, or metabolism [156]. A meta-analysis of antioxidant pharmacological interventions in people revealed that MitoQ was well-tolerated and did not cause any significant side effects [157]. However, MitoQ's therapeutic dosage and manner of delivery require more investigation. Other forms of mitochondrial-targeting antioxidants have garnered acclaim and have been described in excellent reviews [149].

## 7. Conclusion and Perspective

The global prevalence of metabolic inflammation remarkably affects the quality of life of individuals and results in an enormous economic burden. Environmental factors and lifestyle choices are the primary causes of metabolic inflammation, suggesting that it is preventable. Therefore, the pathophysiological mechanism of metabolic inflammation needs to be identified. In this review, numerous pathways of mitochondrial dysfunctionon and the vicious loop between mitochondrial dysfunction and metabolic inflammation are discussed. The altered mitochondrial function was initially designed in response to an energy overload of the body, but the huge metabolic burden can lead to mitochondrial dysfunction, and loss of mitochondrial metabolic buffering capacity and oxidative stress are the basic causes of metabolic inflammation. In terms of organism behavior, mitochondrial dysfunction is closely associated with anxiety [158–160]. Anxiety stimulates the organism's craving for food, especially sweets, which can exacerbate the metabolic burden [161]. The pathological relationship between mitochondrial dysfunction and metabolic inflammation establishes a molecular foundation for the development of mitochondrial-targeted drugs. Researchers have developed or discovered numerous drugs targeting mitochondria (Table 1). However, various physiological barriers hinder targeted organelle delivery of therapeutic drugs, and mitochondria-targeted drug delivery systems have become a great challenge. Currently, mitochondria-targeted drug delivery systems have been extensively developed, including mitochondria-targeted signal peptide, dequalinium, MITO-Porter, TPP, and nanocarriers [162]. Damaged mitochondria can also be replaced with healthy mitochondria, which can be achieved by the formation of gap junction channels between MSC and damaged cells [163]. The development of these therapeutic strategies brings a bright prospect for the treatment of metabolic inflammation targeting mitochondria.

## Figures and Tables

**Table 1:** Therapy agents in metabolic inflammation target mitochondria.

Disease	Agent	Main action	Animal/cell	Signal pathways	Refs
Obesity	Midi-1	Inhibition of Drp1-mediated mitochondrial fission	Ob/Ob mice	AMPK/PGC-1*α*	91
	Resveratrol	Ameliorate oxidative stress	Aged rat fed with HFD	PKA/LKB1/AMPK	108
	MitoQ	Ameliorate oxidative stress	Rat fed with HFD	—	151
	Melatonin	Promote mitochondrial biogenesis	3T3-L1 preadipocytes	—	77
	Capsaicin	Restrict mitochondrial calcium overload	Mice fed with HFD	AMPK/SIRT3	52
	BAM15	Mitochondrial uncoupling	Mice with diet-induced obesity	AMPK	25
	SHC517	Mitochondrial uncoupling	Mice with diet-induced obesity	—	26
Insulin resistance	Melatonin	Improve mitochondrial respiration and biogenesis	Skeletal muscle cell	CREB/PGC-1*α*	78
	Midi-1	Regulation of mitochondrial dynamics	Rat fed with HFD	—	88
	Resveratrol	Ameliorate oxidative stress	Mice fed with HFD	—	109
	MitoQ	Ameliorate oxidative stress	Rat fed with HFD	—	151
	BAM15	Mitochondrial uncoupling	Mice fed with western diet	—	27
	BC1618	Promote mitochondria fission and autophagy	Mice with diet-induced obesity	AMPK	57
T2DM	Midi-1	Inhibited Drp-1-dependent mitochondrial fission	Ob/Ob mice	AMPK/PGC-1*α*	91
	Melatonin	Maintain mitochondrial quality control	Rat with T2D	SIRT6-AMPK-PGC-1*α*-AKT	58
	Resveratrol	Ameliorate oxidative stress and promote mitochondrial biogenesis	Rat with T2D	SIRT1/PGC-1*α*	79
	Metformin	Regulation of mitochondrial dynamics	Leukocytes of patients with T2D	—	89
	MitoQ	Ameliorate oxidative stress	Leukocytes/endothelium of patients with T2D	NF*κ*B	153
Atherosclerosis	MitoQ	Ameliorate oxidative stress	ApoE^–/–^ mice	—	150
	Resveratrol	Maintain mitochondrial homeostasis	HUVECs	AMPK/HIF1/Bnip3	59
	Melatonin	Induce mitophagy and scavenge ROS	ApoE^–/–^ mice	Sirt3/FOXO3a/Parkin	60
	Metformin	Inhibition of Drp1-mediated mitochondrial fission	ApoE^–/–^ mice	AMPK	90
NAFLD	Melatonin	Maintain mitochondrial quality control	Mice fed with HFD	NR4A1/DNA-PKcs/P53	61
	Metformin	Improve oxidative stress and mitochondrial respiration	Hep G2 cell	—	110
	Sorafenib	Mitochondrial uncoupling	Mice fed with HFD	AMPK	28
	Resveratrol	Ameliorate oxidative stress and promote mitochondrial biogenesis	Hep G2 cell	SIRT1/AMPK	80
	Astaxanthin	Ameliorate oxidative stress	Mice fed with HFD	FGF21/PGC-1*α*	111
